# Toward an AI Era: Application of Artificial Intelligence in Inclusion Complex Screening

**DOI:** 10.3390/pharmaceutics18060641

**Published:** 2026-05-23

**Authors:** Naixuan Deng, Yeqi Huang, Yue Gao, Hongluo Li, Wenjing Wang, Minjing Cheng, Chuanbin Wu, Xin Pan, Ling Guo, Junhuang Jiang, Zhengwei Huang

**Affiliations:** 1 State Key Laboratory of Bioactive Molecules and Druggability Assessment, Guangdong Basic Research Center of Excellence for Natural Bioactive Molecules and Discovery of Innovative Drugs, College of Pharmacy, Jinan University, Guangzhou 511443, China; 2 School of Automobile and Transportation Engineering, Guangdong Polytechnic Normal University, No. 293, Zhongshan Avenue West, Tianhe District, Guangzhou 510665, China; 3 School of Pharmaceutical Sciences, Sun Yat-Sen University, Guangzhou 510275, China; 4 Key Laboratory of Tropical Biological Resources of Ministry of Education, School of Pharmaceutical Sciences, Hainan University, Haikou 570228, China; 5 Henan Key Laboratory of Biomarker Detection and Diagnosis for Neurodegenerative Diseases, School of Chemistry and Chemical Engineering, Shangqiu Normal University, Shangqiu 476000, China

**Keywords:** artificial intelligence, machine learning, deep learning, inclusion complexes, rational design, virtual screening

## Abstract

Supramolecular inclusion complexes are widely used in drug delivery and other fields, with the advantages of controllable structures, high stability, excellent biocompatibility, and the ability to improve drug solubility and achieve controlled release. However, traditional screening methods rely on experimental trial and error, which suffer from long cycles, high costs, and low throughput, limiting research and development efficiency. In recent years, the development of artificial intelligence has provided new solutions for the screening of inclusion complexes. This paper systematically reviewed the core technological system of AI in the screening of inclusion complexes, focusing on two aspects: prediction and optimization of key properties and rational design of host molecules, summarizing their specific application progress. At the same time, we analyzed the current core challenges, including data scarcity, insufficient model interpretability, and limited generalization capabilities, and propose future development directions such as building standardized databases, integrating physicochemical principles (e.g., molecular mechanics and thermodynamics), and establishing closed-loop research and development platforms. This review aims to provide a systematic reference for the in-depth application of artificial intelligence in the field of supramolecular inclusion complexes.

## 1. Introduction

Supramolecular inclusion complexes refer to composite systems in which host molecules (such as cyclodextrins, cucurbiturils, crown ethers, calixarenes, and pillararenes) encapsulate guest molecules within their cavities through non-covalent interactions [1]. These complexes have advantages such as controllable structures, high stability, and excellent biocompatibility, making them widely used in areas such as drug delivery and environmental and materials science [2,3,4]. In particular, in the field of drug delivery, they can effectively improve drug solubility, enhance stability, and achieve controlled release [5,6]. For example, the marketed drug Bridion^®^ (Sugammadex) encapsulates rocuronium to reverse neuromuscular blockade [7], Sporanox^®^ (itraconazole) uses hydroxypropyl-β-cyclodextrin to enhance oral bioavailability of a poorly soluble antifungal agent [8], and Abilify^®^ (aripiprazole) employs β-cyclodextrin to improve solubility [9]. These examples illustrate that inclusion complexes are already a proven platform in commercial drug products.

Inclusion complex screening is one of the most important methods to ensure the safety, efficacy, and quality control of drugs. However, the traditional screening of inclusion complexes mainly relies on experimental trial and error, which involves long cycles, high costs, and low throughput, severely limiting the efficiency of research and development in this area [10]. For example, Sapkal et al. reported that the co-precipitation method is quite cumbersome and time-consuming when preparing inclusion complexes on a large scale [11]. In addition, the identification of the optimal carrier often relies on time-consuming and costly microdialysis experiments [12].

**Figure 1:**
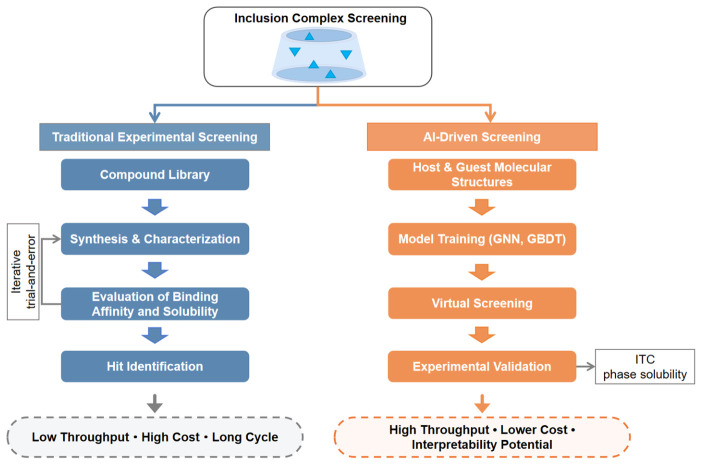
Traditional vs. AI-Driven workflows in inclusion complex screening.

From a drug formulation perspective, the bottleneck is obvious: testing dozens of host-guest pairs experimentally is slow and costly. AI offers a practical alternative by rapidly predicting binding affinity and solubility, allowing formulation scientists to focus experimental work on only the most promising candidates. The core differences between the two screening workflows are shown in Figure 1.

The stability and binding affinity of inclusion complexes are governed by multiple physicochemical factors, including cavity size/shape matching, hydrophobicity, hydrogen bonding and van der Waals interactions, ionization state and pH, stoichiometry, solvent effects, and entropy—enthalpy compensation. These factors collectively determine complexation outcomes and must be considered when designing AI models, as they directly inform the selection of molecular descriptors (e.g., log*P* for hydrophobicity, molecular volume for cavity matching, HBA/HBD counts for hydrogen bonding) and enhance model interpretability.

For pharmaceutical scientists, AI does not replace experiments but reduces unnecessary time and cost, thereby improving early-stage formulation development efficiency. In recent years, with the development of data science, many researchers have attempted to introduce artificial intelligence into the screening process of inclusion complexes. Compared with conventional methods, AI offers advantages such as high-throughput prediction and potentially lower computational cost once the model is trained (while high-quality experimental data generation remains expensive), as well as offering potential for improved interpretability when using techniques such as attention mechanisms, although many deep learning models remain inherently black-box. These capabilities can accelerate the discovery and optimization of inclusion complexes. Specifically, AI can not only achieve high-throughput virtual screening rapidly and accurately but also predict key properties of inclusion complexes, such as stability constants, solubility, and stability, and can even enable the de novo design of novel host molecules [13].

Although AI has gained widespread application in drug discovery, there is still a lack of systematic discussion specifically targeting the screening of inclusion complexes. Therefore, this paper aimed to systematically review the core technologies and application progress of artificial intelligence in the study of inclusion complexes, while critically analyzing the current challenges and future trends, providing a theoretical reference for researchers in related fields.

## 2. Core AI Technologies in Inclusion Complex Research

### 2.1. Representative Artificial Intelligence Algorithms

**Figure 2:**
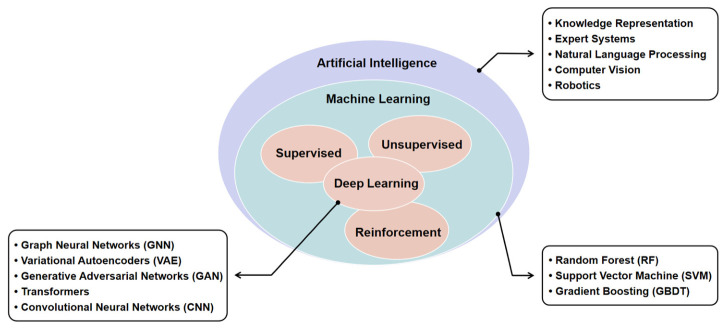
Hierarchical relationship among artificial intelligence, machine learning, and deep learning.

AI aims to enable computers to simulate human intelligent behavior. Its technical system encompasses multiple subfields, including knowledge representation, expert systems, natural language processing, computer vision, and robotics. Among these, machine learning (ML) serves as the mainstream technique for achieving this goal. By training models from data, machine learning allows computers to automatically learn patterns and make predictions or decisions, and it is typically divided into supervised learning, unsupervised learning, and reinforcement learning [14,15]. In the field of inclusion complex screening, these ML methods have been successfully applied to predict host-guest binding affinity, solubility, and stability, offering a powerful alternative to traditional experimental trial and error. Deep learning (DL), an advanced branch of ML based on multi-layer artificial neural networks [16,17], is particularly suitable for handling the complex molecular structures of cyclodextrins, calixarenes, and other macrocyclic hosts. The hierarchical relationship among artificial intelligence, machine learning, and deep learning is shown in Figure 2.

In the study of supramolecular inclusion complex screening, three main types of methods are primarily used depending on the characteristics of the data and the specific task objectives: traditional machine learning, deep learning, and generative models and optimization algorithms for molecular design and process optimization. The features and application scenarios of representative methods are summarized in Table 1.

Traditional machine learning algorithms, such as Gradient Boosting (GBDT) and Support Vector Machines (SVMs), form a robust foundation of this field. These algorithms have relatively simple structures, require a moderate amount of data, and possess good interpretability, typically making them suitable for small-scale datasets [18,19].

Deep learning algorithms are better suited for handling the complex topological structures and spatial relationships inherent in molecules [20]. Common architectures include Convolutional Neural Networks (CNNs), Graph Neural Networks (GNNs), and Transformers. Among these, GNNs are the most widely applied. GNNs enable end-to-end learning of deep features of molecular structures, particularly useful for modeling host-guest cavity interactions in inclusion complexes, reducing dependence on manually designed descriptors [21].

Generative models and optimization algorithms, including Variational Autoencoders (VAEs) [22], Generative Adversarial Networks (GANs) [23], evolutionary algorithms [24], and Bayesian optimization [25], are primarily used to explore novel chemical structures and optimize process conditions. These methods can not only conduct intelligent searches in chemical space to generate new host molecules with potential inclusion capabilities but also optimize the preparation parameters of inclusion complexes, such as the feed ratios and temperature, achieving the inverse design from target properties to molecular structures [26,27].

**Table 1:** Representative AI algorithms and their characteristics in inclusion complex screening.

Algorithm Category	Representative Models	Core Principle	Limitations	Application Scenarios	References
Supervised Learning	Random Forest (RF)	Ensemble of decision trees for regression/classification	Overfitting on noisy data; limited capacity for complex molecular features	Predicting binding affinity between cyclodextrins and poorly soluble drugs	[28,29]
	Gradient Boosting (XGBoost, LightGBM)	Sequential ensemble correcting previous errors	Sensitive to hyperparameters; computationally intensive	High-precision regression tasks (Δ*G*, log*P*)	[30,31]
	Support Vector Machine (SVM)	Maximizes margin between classes via kernel trick	Inefficient on large datasets; kernel selection matters	Small-to-medium dataset classification and regression	[32,33,34]
	Graph Neural Networks (GNNs)	Message passing on atom-bond graphs to learn molecular topology	Computationally expensive; interpretability limited	Learning host-guest interactions directly from molecular graphs of cyclodextrin-drug pairs	[21,35]
Unsupervised/Generative Models	Variational Autoencoder (VAE)	Learns latent distribution of molecular structures for generation	Prone to posterior collapse, reducing generative power; limited latent space disentanglement, hindering property-controlled molecular design	De novo molecules design	[36]
	Generative Adversarial Networks (GANs)	Adversarial training to generate novel molecular structures	mode collapse and unstable training	Exploration of chemical space for new host-guest systems	[23,37]
Optimization Algorithms	Bayesian Optimization	Probabilistic surrogate model for global optimization of expensive functions	Scales poorly to high-dimensional spaces; prior selection matters	Optimization of experimental conditions (e.g., host-guest ratio, temperature)	[25]

### 2.2. Molecular Characterization Methods

Molecular characterization is one of the most important factors affecting the performance of AI models [38,39]. The molecular structure determines its physicochemical properties, and different molecular representations directly influence whether the model can effectively extract structural information relevant to the prediction target. Appropriate representations can fully preserve the structural information of molecules, thereby improving prediction accuracy. The evolution has progressed from linear symbols to mathematically designed descriptors and further to advanced representations capable of autonomously learning complex features [40].

In the early stage, the most widely used methods were Simplified Molecular Input Line Entry Specification (SMILES) and various molecular fingerprints. SMILES is a linear string that describes molecular topology and atomic connectivity using American Standard Code for Information Interchange (ASCII) characters. AI models can directly read SMILES strings and convert them into internal feature representations or use them to generate entirely new molecular structures, supporting drug design and similarity analysis [41]. Molecular fingerprints encode molecular structures into fixed-length bit strings, extracting substructure features through predefined rules, essentially serving as binary vectors. Commonly used fingerprints include Extended Connectivity Fingerprints (ECFP), Molecular ACCess System fingerprints (MACCS), and PubChem fingerprints [42,43]. They have been widely used for compound activity prediction, virtual screening, and similarity searches, accelerating the drug discovery process.

In recent years, graph-based molecular representations have become the mainstream approach. Compared to linear SMILES strings and molecular fingerprints, graph representations can more comprehensively encode the topological and spatial information of molecules. They abstract molecules as graph structures with atoms as nodes and chemical bonds as edges. Combined with deep learning architectures such as GNNs and Message Passing Neural Networks (MPNNs), they can directly learn deep feature representations of molecules, enabling various property prediction tasks. For inclusion complexes, graph-based representations are especially advantageous because they naturally capture the cyclic topology of host molecules (e.g., cyclodextrins) and can explicitly model non-covalent interactions between host and guest atoms.

**Figure 3:**
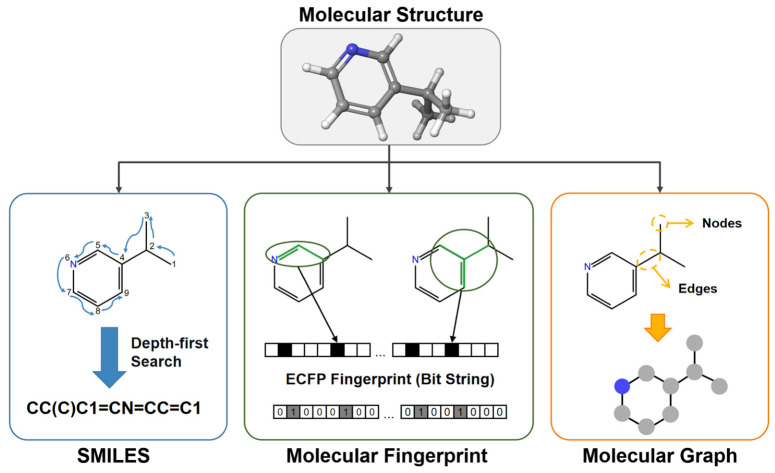
From Structure to SMILES, Fingerprint, and Graph.

The conversion of a molecular structure into computer-readable representations is illustrated in Figure 3, using 3-propan-2-ylpyridine as an example. SMILES is generated by depth-first search of the molecular skeleton, molecular fingerprints encode structural fragments into bit strings, and the molecular graph maps atoms and bonds to nodes and edges, respectively.

### 2.3. Ad Hoc Tools

The deep integration of artificial intelligence and pharmaceutical research has given rise to a series of integrated web platforms that can be used for the screening and design of preparations such as inclusion complexes.

The FormulationAI platform developed by Dong et al. [44] is the first free and comprehensive web-based tool for formulation design. The platform integrates the most extensive datasets covering six drug formulation systems, including cyclodextrin formulations. During model development, the platform systematically compared multiple machine learning algorithms, such as RF, SVM, LightGBM, and XGBoost, combined with molecular descriptors and molecular fingerprints, achieving intelligent prediction of 16 key properties. Users can quickly achieve formulation design by simply inputting basic information about the drug and excipients (https://formulationai.computpharm.org/, accessed on 5 May 2025).

FormulationDT developed by Wang et al. [45] is the first data-driven and knowledge-guided AI platform for formulation strategy design. This platform learns from approved drug formulations and constructs a comprehensive system covering 12 decisions for oral and injectable administration. To address the positive-unlabeled (PU) data scenario, the platform uses the PU-Decide framework, which utilizes base classifier selection and label recovery strategies, with sub-classifiers built using decision trees, SVMs, k-nearest neighbors, and logistic regression. This approach yields high-precision and interpretable classification models with ROC_AUC scores ranging from 0.78 to 0.98 (http://formulationdt.computpharm.org/, accessed on 5 May 2025).

Although these platforms are primarily developed for formulation development, their methodologies offer valuable references for the virtual screening and rational design of inclusion complexes.

## 3. Application of Artificial Intelligence in Inclusion Complex Screening

**Figure 4:**
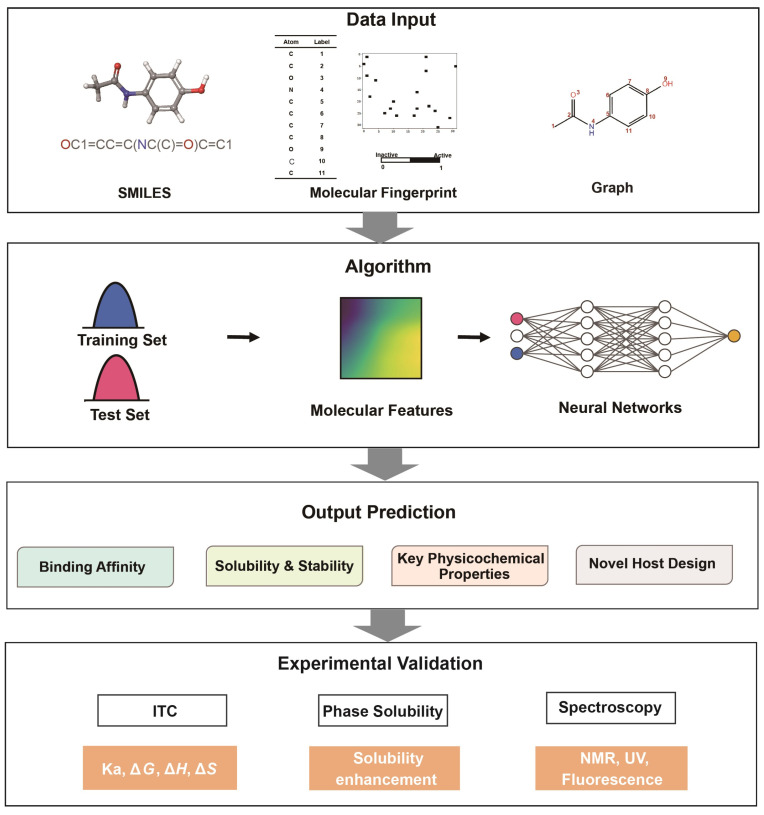
AI-driven workflow for inclusion complex screening.

The AI-driven workflow typically includes molecular data input, feature extraction, and model training, ultimately generating predictive outputs, as summarized in Figure 4. AI is applied at various stages of drug development, from predicting binding affinity and key physicochemical properties to enabling high-throughput virtual screening and even de novo design of novel host molecules, comprehensively enhancing the efficiency of drug discovery.

### 3.1. AI-Based Property Prediction and Screening for Inclusion Complexes

A fundamental task in inclusion complex research is the accurate prediction of binding affinity between host and guest molecules. Achieving this requires large-scale, high-quality binding affinity datasets for AI model learning. However, for cyclodextrin-based inclusion complexes, especially those involving poorly soluble molecules such as cholesterol, experimental determination of binding constants often faces challenges such as poor reproducibility and high costs, which severely limit the scale and quality of the data. To address this, Anderson and colleagues developed a fully automated workflow based on metadynamics simulations and mean force potential calculations for efficiently and accurately determining the 1:1 inclusion free energy (Δ*G*) and binding constant (*K_a_*) of various β-cyclodextrin derivatives with sterol molecules. They also proposed a “broad-sampling” strategy that significantly reduces computational cost while ensuring accuracy. This work provides a scalable solution for generating standardized, mechanistically clear affinity data, laying a data foundation for AI model training [46].

Based on such data, various machine learning models have been successfully applied to predict binding affinities of inclusion complexes. Ye et al. collected approximately 3000 data points and developed a model to predict the binding free energy of cyclodextrin-guest molecule complexes, systematically comparing the performance of algorithms including LightGBM, random forest, and deep learning. The results showed that LightGBM achieved the best prediction accuracy, with a mean absolute error of 1.38 kJ/mol and a coefficient of determination *R*^2^ of 0.86 [47]. Similarly, Carvalho et al. evaluated three machine learning methods—Gaussian Process Regression (GPR), Support Vector Regression (SVR), and eXtreme Gradient Boosting (XGBoost)—on a dataset of 280 cyclodextrin-guest complexes from the BindingDB database. Using 21 molecular descriptors as input features and optimizing hyperparameters via random search, the GPR model achieved an *R*^2^ of 0.803 and an RMSE of 1.811 kJ/mol on the test set, further confirming the applicability of machine learning for affinity prediction in cyclodextrin-based systems [48]. Ma and colleagues established a balanced dataset containing 200 experimentally verified compounds (100 positive and 100 negative samples). Using molecular structure descriptors and solvation free energy quantified by molecular dynamics simulations as input features, they built artificial neural network, SVM, and logistic regression models to predict the likelihood of guest molecules forming inclusion complexes with β-cyclodextrin [49]. To address the issue of variable performance in accuracy and recall for individual models, the study further designed recall-priority and precision-priority strategies, providing a feasible approach for machine learning-assisted virtual screening of inclusion complexes.

The application of machine learning methods in host-guest chemistry has also expanded to other systems. The recently reported “CrownBind-IA” model successfully achieved high-precision prediction of binding constants between crown ethers and alkali metal ions using machine learning algorithms, with *R*^2^ > 0.95 [50]. Furthermore, one study employed an SVM to predict the binding affinity of small molecules with cucurbit[7]uril (CB[7]) and experimentally validated the model’s predictions for the drugs TAK-580 and ceritinib. The study also found that the homolog CB[8] exhibited binding preference for selumetinib, suggesting that drug release could be modulated by adjusting the ratio of CB[7] to CB[8], further highlighting the value of machine learning in the design of supramolecular drug delivery systems [51].

In addition to binding affinity, AI can predict other key properties that are critical for formulation development, such as solubility and stability. Merzlikine et al. used Cubist and random forest algorithms to build QSPR models that predict cyclodextrin inclusion free energy, which can be used to predict the solubilization effects of cyclodextrins on drugs and provide quantitative guidance for early formulation screening [52]. In recent years, deep learning methods such as GNNs have further advanced the predictive capabilities for molecular properties. Related models can be directly transferred to supramolecular systems to predict key parameters including apparent solubility and complex stability constants upon guest inclusion [53,54]. Meanwhile, the evaluation metric systems adopted in generative models, such as Quantitative Estimation of Drug-likeness (QED) and Synthetic Accessibility score (SA score), also provide quantitative tools for assessing the drug-likeness and synthetic feasibility of inclusion complexes [55,56].

AI techniques can also be applied to multi-objective optimization of complex formulation systems containing inclusion complexes. Suriyaamporn et al. employed various machine learning algorithms to predict the swelling and erosion rates of drug-loaded hydrogel microneedles, optimizing preparation parameters and establishing an “AI prediction-experimental validation” closed-loop development cycle [57]. Additionally, Zhang et al. utilized artificial neural networks combined with genetic algorithms to globally optimize the preparation parameters of Baeckeae oil-β-cyclodextrin inclusion complexes. The optimized conditions significantly improved the oil loading and inclusion yield, demonstrating the feasibility of the “ANN modeling-GA optimization” strategy in formulation process optimization.

Beyond algorithm selection, proper validation strategies are critical for developing reliable AI models for inclusion complex screening. Cross-validation (e.g., 5-fold or 10-fold) is commonly used for small datasets, while an independent test set is preferred for assessing generalization performance [58]. However, random splitting may cause data leakage when similar host or guest molecules appear in both training and test sets; scaffold-based or cluster-based splitting is recommended to avoid overly optimistic estimates [59]. The applicability domain of a model should be defined to flag molecules too dissimilar from the training set [60]. Uncertainty quantification techniques, such as Monte Carlo dropout or ensemble variance, can provide confidence intervals for predictions, helping experimentalists prioritize candidates for validation [61]. Host-guest pairs experimentally confirmed not to form complexes are referred to as negative data. They are often missing from existing databases but are critical for avoiding false-positive predictions [62]. Without these practices, AI models for inclusion complexes risk poor generalizability across different experimental systems.

### 3.2. Rational Design of Host Molecules

Generative AI represents an emerging direction for the rational design of host molecules, though it currently faces substantial challenges. By learning the intrinsic relationships between molecular structure and function through deep learning algorithms, generative models may eventually enable the targeted generation of host molecules with specific cavity sizes to optimize drug delivery performance. However, macrocyclic host molecules present unique challenges for generative models compared to ordinary small molecules, including ring closure constraints, cavity geometry control, and synthetic feasibility [63]. Most published generative models have been validated on small drug-like compounds rather than macrocyclic hosts.

At the molecular representation level, chemical language models lay the foundation for handling the structures of target molecules. Self-Referencing Embedded Strings (SELFIES), as a robust molecular string representation, have a decoder that automatically ignores symbols that would violate valence rules, allowing generative models to explore chemical space without learning complex grammar in advance [64]. Molecular graph representation treats atoms as nodes and chemical bonds as edges, processing molecular topology directly through GNNs, offering a more natural representation for structural modification of host molecules [22].

At the generation strategy level, fragment-based assembly methods effectively improve the structural rationality of generated molecules. Recurring fixed atomic combinations, such as methyl groups, carboxyl groups, and benzene rings, can serve as building units, a strategy particularly suitable for the design of large molecules [65]. In supramolecular systems, host molecules such as cyclodextrins can be broken down into sugar unit fragments, and these fragments can be assembled according to the molecular size and polarity of the target drug to generate novel host molecules with cavity-adaptive properties. Conditional Variational Autoencoders (Conditional VAEs) and Transformer-based conditional generative models (cTransformer) further enable target-constrained generation, establishing a mapping between drug molecular features and host molecular structures [66].

In terms of multi-constraint optimization, host design must simultaneously satisfy multiple requirements including cavity matching, synthetic feasibility, and solubilization efficacy. Lavecchia systematically summarized physicochemical property metrics used to evaluate the quality of generated molecules, including molecular weight, log*P*, topological polar surface area, etc., as well as SA scores and QED [55,56]. In inclusion complex research, these metrics can be translated to assess the drug-likeness of host-drug complexes, with QED indicating whether inclusion improves drug-likeness deficiencies and SA evaluating the synthetic difficulty of host derivatives. Common optimization methods include gradient descent in latent space, Pareto optimization, and reinforcement learning [67,68,69].

In summary, generative AI for host design is an emerging field. Most published models have been validated on small drug-like compounds, not macrocyclic hosts, and substantial methodological progress is still required for practical application.

## 4. Limitations

Although AI has shown great potential in inclusion complex screening, its practical application still faces a series of core challenges, specifically including the following three aspects:

(1) Data scarcity and insufficient standardization. High-quality, standardized experimental datasets are still scarce. On the one hand, existing molecular datasets such as ChEMBL and ZINC are mainly aimed at drug discovery, lacking proprietary data specific to supramolecular inclusion complex systems. On the other hand, a large amount of experimental data is scattered across different institutions or locked behind proprietary walls within pharmaceutical companies, limiting access to a comprehensive labeled corpus and consequently constraining the training effectiveness of deep learning models [70,71].
(2) Weak model interpretability. Complex models, particularly deep learning systems, often have “black box” characteristics, making the decision logic in the molecular design process difficult to trace. Even if key features are identified, the relationship between these features and the prediction results may be indirect and unstable, and even small perturbations can change the model’s output [72]. Moreover, while techniques such as attention mechanisms and feature importance analyses can provide some degree of interpretability, they do not fully resolve the black-box nature of deep learning, and their explanations should be validated against physicochemical principles. This lack of transparency limits the scientific validation of host-guest interaction mechanisms and hinders the application of AI methods in rigorous scientific validation scenarios [73].
(3) Insufficient model generalization. A model trained on one system is often difficult to directly transfer to other systems. For example, in the SAMPL4 host-guest blind challenge, a density function theory-based method performed excellently in predicting the binding affinity of the cucurbit[7]uril systems, with a coefficient of determination between predicted and experimental values as high as 0.8. However, the same method performed poorly for octa-acid systems, with a coefficient of determination of only 0.4, below the average level [74].

## 5. Future Recommendations

**Figure 5:**
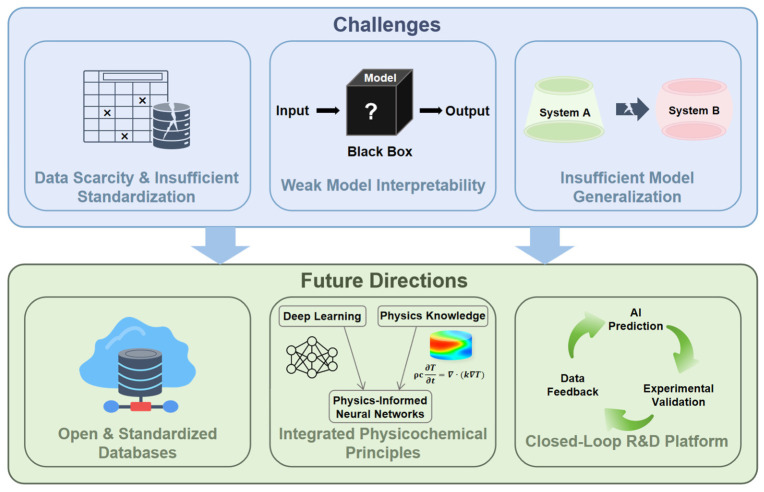
Challenges and future directions in AI-driven inclusion complex screening.

The current challenges and future directions of AI-driven inclusion complex screening are summarized in Figure 5. We then discuss the corresponding solutions for the three major challenges mentioned above.

(1) Establish open and standardized databases. To enable effective AI model training, such databases should systematically collect not only host-guest binding constants and inclusion ratios but also detailed experimental conditions (including temperature, pH, buffer composition), thermodynamic parameters (e.g., free energy change Δ*G*, enthalpy change Δ*H*, and entropy change Δ*S*), and solubility data. In addition, negative samples that fail to form inclusion complexes should also be recorded to avoid over-prediction or false positive results. By constructing a dataset with complete information, the errors introduced by data heterogeneity from different sources can be significantly reduced, thereby improving the data quality and reliability for AI model training.
(2) Integrate physicochemical principles into model design. By introducing knowledge from molecular mechanics and thermodynamics, physics-informed neural networks can be constructed to enhance the scientific validity of model predictions.
(3) Build a closed-loop research and development (R&D) platform featuring “AI prediction-experimental validation-data feedback”, connecting generative models, predictive models, and automated experimental platforms to form a continuous iterative optimization cycle.

Furthermore, beyond traditional machine learning and deep learning, we can also use knowledge graph to systematically integrate the relationships between host molecular structures and cavity characteristics while leveraging symbolic AI to enhance the model’s logical reasoning capability, improve the model’s interpretability of complex inclusion complex formation mechanisms, and promote the screening of inclusion complexes toward a more intelligent and interpretable direction. Also, in addition to inclusion complex, it is reasonable that AI can be employed in the (pre)formulation processes of more drug delivery systems. We anticipate a boost in relevant studies in the foreseeable future.

## 6. Conclusions

AI is transforming the research paradigm of inclusion complex screening and design, showing great potential in multiple fields. By using machine learning models to predict binding affinity, employing generative models to design novel carrier molecules, and leveraging optimization algorithms, AI has significantly enhanced the research efficiency of inclusion complexes. However, to fully harness the capabilities of AI, challenges such as data quality issues, insufficient model interpretability, and poor generalization still need to be overcome. In the future, with the establishment of standardized databases, deeper integration of physicochemical principles, and the improvement of prediction-validation closed-loop platforms, artificial intelligence is expected to achieve broader applications in the field of supramolecular inclusion complexes, providing more reliable tools for the rational design of drug delivery systems. In practical terms, formulation scientists can use AI to prioritize which host-guest pairs to test, shortening development timelines and reducing costly trial and error.

## Data Availability

No new data were created or analyzed in this study. Data sharing is not applicable to this article.
